# Der Wirtschaftsstabilisierungsfonds — eine (Zwischen-)Bilanz

**DOI:** 10.1007/s10273-022-3136-1

**Published:** 2022-03-19

**Authors:** Philipp Steinberg, Holger Lüthen, Lukas Gehring, Daniel Schulz-Bianco

**Affiliations:** 1 Abteilung Wirtschaftspolitik, Bundesministerium für Wirtschaft und Energie, Scharnhorststr. 34-37, 10115 Berlin, Deutschland; 2 Abteilung Wirtschaftspolitik, Referat IE3, Bundesministerium für Wirtschaft und Energie, Scharnhorststr. 34-37, 10115 Berlin, Deutschland

**Keywords:** H12, H81, H84

## Abstract

Der Wirtschaftsstabilisierungsfonds (WSF) ist eines von mehreren staatlichen Unterstützungsinstrumenten, die zur Bekämpfung der Folgen der Coronakrise aufgelegt wurden. Der WSF wurde für Unternehmen mit besonderer (volks-)wirtschaftlicher Bedeutung konzipiert. Dabei verfügt der Fonds über ein breites Spektrum an eigenkapitalähnlichen Hybridinstrumenten, wie stillen Beteiligungen und Nachrangdarlehen, wobei im Ausnahmefall auch offene Beteiligungen eingesetzt werden. Trotz der geringen Zahl stabilisierter Unternehmen zeigt sich, dass der WSF ein wichtiger Baustein der Krisenarchitektur ist — dank passgenauer Stabilisierungslösungen konnten wichtige Unternehmen erfolgreich stabilisiert werden.

## Abstract (en)

The Economic Stabilisation Fund (ESF) is one of several German state aid instruments set up to combat the economic fallout of COVID-19. The ESF offers a range of instruments from equity to hybrid capital, including silent participation and subordinated loans. In exceptional cases, the ESF may also become a direct shareholder. While the ESF stabilised only a relatively small number of companies, it constitutes an important building block in Germany’s overall business support framework. Due to its tailor-made stabilisation measures, companies of economic significance were successfully stabilised during the pandemic.

Um massenhafte Insolvenzen zu verhindern, Arbeitsplätze und gleichzeitig das Vertrauen der Wirtschaft und der Bürger:innen in die Politik zu sichern, wurde seit Pandemiebeginn ein umfassendes Paket staatlicher Rettungsmaßnahmen geschnürt und kontinuierlich weiterentwickelt. Jenseits von steuerlichen Maßnahmen und Kurzarbeitergeld können Unternehmen rückzahlungspflichtige und nicht rückzahlungspflichtige Hilfen in Anspruch nehmen.[1] Zuschussprogramme wie die Überbrückungshilfen gewähren Mittel bis zu einer Obergrenze von 54,5 Mio. Euro[2] je nach Höhe des Umsatzausfalls. Bis Ende 2021 wurden insgesamt über 61 Mrd. Euro an nicht rückzahlungspflichtigen Hilfen ausgezahlt. Bei den rückzahlungspflichtigen Maßnahmen spielen neben den (Groß-)Bürgschaften des Bundes und der Bürgschaftsbanken auch Finanzierungen durch das KfW-Sonderprogramm eine wichtige Rolle. In diesem Rahmen wurden bis Ende 2021 Kredite über 55 Mrd. Euro zugesagt. Die Mittel zielen mit günstiger, teilweise risikounabhängiger Verzinsung auf eine rasche Deckung des Liquiditätsbedarfs ab. Die Maßnahmen richten sich vornehmlich an Unternehmen und freiberuflich Tätige der gewerblichen Wirtschaft. Die überwiegende Mehrzahl der Antragstellenden (98 %) sind dabei kleine und mittlere Unternehmen (KMU) mit Antragsvolumina bis 100.000 Euro (Dashboard Deutschland, 2022).

Im Gegensatz dazu sind größere oder komplexere Bedarfe mit standardisierten Hilfsprogrammen nur schwer adressierbar – Maßnahmen dieser Art müssen im Einzelfall geprüft, genehmigt und individuell angepasst werden. Diese Fälle übernimmt der Wirtschaftsstabilisierungsfonds (WSF). Mit einer maximalen Ausstattung von 600 Mrd. Euro stellte der WSF das potenziell größte Unterstützungsinstrument Deutschlands dar.[3] Eine individuelle Bearbeitung jedes Antrags soll eine maßgeschneiderte Lösung sicherstellen. Der Fonds, der ausschließlich rückzahlungspflichtige Maßnahmen gewährt, hat das gesetzgeberische Ziel, betroffenen Unternehmen zu helfen, ihre Kapitalbasis zu stärken und Liquiditätsengpässe zu überwinden. Hierbei wurde der WSF als letzter Rettungsanker für Unterneh-

1 Für eine Übersicht und bisher gewährte Volumina der Programme siehe Dashboard Deutschland (2022).

2 Fördergrenze für Überbrückungshilfe III, III Plus und IV, soweit die Antragstellerin keine Beihilfen aus anderen staatlichen Corona-Förderprogrammen auf Basis der einschlägigen Beihilferahmen erhalten hat (FAQ Überbrückungshilfe, 2022).

* An dem Artikel haben weitere Mitarbeiter:innen des BMWK mitgewirkt. Wir danken Lennert Althoff, Jennifer Jeutner, Stefanie Jungheim-Herwig, Larissa Karthaus, Annick Moiteaux, Dirk Neumann, Stefan Sawatzki, Jochen Schaller, Thomas Solbach und Ulrike Zirpel.

3 Dieses Volumen wurde im Zuge der Verlängerung des WSF bis zum 30.6. 2022 auf 400 Mrd. Euro reduziert.

**Dr. Philipp Steinberg** ist Abteilungsleiter Wirtschaftspolitik im Bundesministerium für Wirtschaft und Klimaschutz (BMWK) sowie Ressortkoordinator Nachhaltigkeit.

**Dr. Holger Lüthen, Lukas Gehring** und **Dr. Daniel Schulz-Bianco** sind dort Referenten im Referat „Rekapitalisierungen, Monitoring und Beendigung Stabilisierung“ in der Abteilung Wirtschaftspolitik.

men konzipiert, d.h. es dürfen im konkreten Fall keine alternativen Finanzierungsmöglichkeiten zur Verfügung stehen (§ 25 Abs. 1 S. 1 Stabilsierungsfondsgesetz). Eine Kombination von WSF-Maßnahmen mit anderen öffentlichen Corona-Hilfsprogrammen ist dennoch möglich und üblich, sofern die Zuschüsse der anderen Programme nicht ausreichen. Ebenso gelten beihilferechtliche Kumulierungsregeln: Um sicherzustellen, dass nur der tatsächliche Liquiditätsbedarf gedeckt wird, werden anderweitige Unterstützungsleistungen bei den WSF-Anträgen berücksichtigt bzw. angerechnet. Die Rolle des WSF als „lender of last resort“ bleibt damit stets gewahrt.

Bis zum 31.12.2021 wurden Rekapitalisierungen im Gesamtvolumen von rund 8,8 Mrd. Euro schwerpunktmäßig in Form von Nachrangdarlehen und stillen Beteiligungen gewährt.[4] Einzig bei der Lufthansa ist der WSF am Unternehmen direkt beteiligt (derzeit Aktienbeteiligung von rund 14,1 %), zudem hält der WSF bei TUI ein Wandlungsrecht, das eine Aktienbeteiligung von über 20 % ermöglicht. Bis dato hat der WSF 21 volkswirtschaftlich bedeutende Unternehmen mit insgesamt mehreren hunderttausend Arbeitsplätzen in der Krise stabilisiert.

## Institutionelle Grundlage

Die politische Situation zu Beginn der Pandemie war ein Moment radikaler (oder Knightscher) Unsicherheit (Knight, 1921).[5] Solche Situationen, die ohne jüngeres historisches Beispiel und damit in hohem Maße einzigartig sind, stellen politische Akteure vor ein Dilemma. Einerseits stehen sie unter akutem Handlungsdruck, andererseits macht es die Einzigartigkeit der Situation unmöglich, eine abschließende Liste potenzieller Szenarien aufzustellen und diesen Szenarien Wahrscheinlichkeiten zuzuordnen. Mathematisch sind die mit ihren Entscheidungen verbundenen Risiken erst recht nicht kalkulierbar. Politische Akteure sehen sich daher gefordert, Strategien zu entwickeln, diese Unsicherheit zu verringern. Eine naheliegende Option stellt dabei der Rückgriff auf Konzepte und Instrumente dar, die sich in vergangenen Krisensituationen bewährt haben. Damit kann ein hohes Maß an Unsicherheit zur Pfadabhängigkeit politischer Entscheidungen beitragen. Das heißt, dass positive Feedback-Effekte einer vergangenen Entscheidung einen einmal eingeschlagenen Pfad in (sozialen) Prozessen immer weiter verstärken (North, 1990; Pierson, 2004). In der Politik sind positive Feedback-Effekte unter anderem in der Logik kollektiven Handelns, hoher Komplexität sowie der starken Institutionalisierung vergangener Entscheidungen durch Gesetze und Rechtsverordnungen begründet, die künftige Handlungsspielräume einengen. Hohe Komplexität und Unsicherheit verstärken zudem die Tendenz politischer Akteure, neue Information im Lichte bereits bestehender „mental maps“ (kognitiver Karten) zu interpretieren, was pfadabhängige Entscheidungen begünstigt.

Entsprechend baute der WSF auf die Erfahrungen und Strukturen der Finanz- und Wirtschaftskrise 2008 auf. So wurde das Rahmenwerk des Finanzmarktstabilisierungsfondsgesetzes (FMStFG) von 2008 an die Besonderheiten der Realwirtschaft und der Coronapandemie angepasst. Dieser Rückgriff ermöglichte eine rasche Umsetzung und Abstimmung mit allen Akteuren. Das gesetzgeberische Verfahren, das in das Stabilisierungsfondsgesetz (StFG) mündete, konnte dadurch bereits im März 2020 abgeschlossen und die behilferechtliche Genehmigung des WSF durch die EU-Kommission im Juli 2020 erreicht werden.

Der Rückgriff erklärt auch das potenziell große Gesamtvolumen des WSF, welches im Nachhinein leicht als „überdimensioniert“ bezeichnet werden könnte. Die Erfahrung aus der globalen Finanzkrise hat jedoch gezeigt, dass es sinnvoll ist, einen Stabilisierungsfonds mit umfangreichen Mitteln auszugestalten, um auch bei einer Verschärfung der Krise Handlungsspielraum zu erhalten. Schließlich muss auch der Optionswert des WSF bedacht werden, da er als „lender of last resort“ ohnehin nur nachranging tätig wird, d. h. dann, wenn alle alternativen Finanzierungsmöglichkeiten bereits ausgeschöpft sind. Ein zu kleiner Fonds könnte dieser Rolle nur schwerlich gerecht werden.

## Charakteristika des WSF

### Zugangskriterien

Der WSF wurde für Unternehmen mit besonderer (volks-)wirtschaftlicher Bedeutung konzipiert, wobei er explizit für den (größeren) Mittelstand offengehalten wurde (§ 16 Abs. 2 und 1 StFG).[6] Diese Bedeutung muss in einer ausführlichen volkswirtschaftlichen Prüfung durch das Bundesministerium für Wirtschaft und Klimaschutz (BMWK) bestätigt werden. Konkret müssen antragstellende Unternehmen in den letzten beiden bilanziell abgeschlossenen Geschäftsjahren vor dem 1. Januar 2020 mindestens zwei der drei sogenannten WSF-Zugangskriterien erfüllen: mehr als 43 Mio. Euro Bilanzsumme, mehr als 50 Mio. Euro Umsatzerlöse und mehr als 249 Beschäftigte. Für kleinere Unternehmen und Start-ups ist der WSF dann zugänglich, wenn sie eine besondere Bedeu-

4 Stand Januar 2022, aktuelles Volumen der Maßnahmen unter https://www.deutsche-finanzagentur.de/de/wirtschafts-stabilisierung/.

5 Das Konzept spielte eine Rolle bei John Maynard Keynes, führte ansonsten aber bis zur Finanzkrise 2008 eher ein Schattendasein in der (polit-)ökonomischen Forschung. Seither hat es an neuer Prominenz gewonnen.

6 Beim WSF wird die Mittelstandsdefinition der KfW zugrunde gelegt. Demnach werden alle Unternehmen unabhängig von der Beschäftigtenzahl mit einem Jahresumsatz von bis zu 500 Mio. Euro als mittelständische Unternehmen bezeichnet.

tung für die Sicherheit oder die Wirtschaft haben, z. B. viel genutzte Software-Entwicklungen oder Cloud-Computing-Dienste. Startups müssen überdies von privaten Kapitalgebern mit einem Unternehmenswert von mindestens 50 Mio. Euro bewertet worden sein. Der WSF-Ausschuss[7] entscheidet über solche Ausnahmefälle gesondert.

Antragstellende Unternehmen müssen eine klare eigenständige Fortführungsperspektive nach Überwindung der Pandemie vorweisen und sich vor Pandemiebeginn nicht in Schwierigkeiten befunden haben.[8] Bei der Entscheidung über die Stabilisierungsinstrumente wird zudem die Bedeutung des Unternehmens für die Wirtschaft, die Dringlichkeit, die Auswirkungen auf Arbeitsmarkt und Wettbewerb sowie der Grundsatz eines möglichst sparsamen und wirtschaftlichen Mitteleinsatzes geprüft (§ 20 Abs. 1 StFG). Die Ausgestaltung der Instrumente ist in der Verordnung zur Gewährung und Durchführung von Maßnahmen aus dem Wirtschaftsstabilisierungsfonds nach dem Stabilisierungsfondsgesetz (WSF-DV) geregelt und muss die Maßgaben der beihilferechtlichen Genehmigung der EU-Kommission einhalten.

## Gestaltung der Stabilisierungsmaßnahmen

Der unternehmensspezifische Bedarf zur Vermeidung einer coronabedingten Insolvenz und zur Wiederherstellung der Rentabilität ist sehr unterschiedlich, wie sich insbesondere in den Volumina gewährter WSF-Einzelmaßnahmen zeigt – von einstelligen Millionen- bis hin zu Milliardenbeträgen. Eine passgenaue Stabilisierung im Einklang mit haushälterischen Grundsätzen und beihilferechtlichen Auflagen erfordert eine sorgsame Ausgestaltung von Instrumenten und Konditionen auf Einzelfallbasis in enger Abstimmung mit den Unternehmen.

Als Stabilisierungsmaßnahmen verfügt der WSF über ein breites Spektrum an eigenkapitalähnlichen Hybridinstrumenten, wie stillen Beteiligungen und Nachrangdarlehen, wobei im Ausnahmefall auch offene Beteiligungen eingesetzt werden. Die Maßnahmen kommen dann in Betracht, wenn bei krisenbedingtem Verlust von Eigenkapital die Zufuhr von Nachrangkapital oder Eigenkapital erforderlich ist, um die Kreditfähigkeit wiederherzustellen. Zudem kann der WSF Gewährleistungen für verschiedene Fremdkapitalinstrumente übernehmen (insbesondere Garantien), auch wenn dies in der Praxis bislang nicht vorkam.

Generell erhebt der WSF eine marktübliche Verzinsung auf die rückzahlungspflichtige Stabilisierung. Die Zinsen setzen sich aus instrumentenspezifischen Marktzinsen und unternehmensspezifischen Risikoindikatoren zusammen. Konkret werden dabei Auf- und Abschläge zu den beihilferechtlich vorgegebenen Mindestzinsen erhoben, die Verschuldungsgrad, Geschäftsmodell, Zukunftsperspektive sowie finanzielle Beiträge zur Stabilisierung durch die Gesellschafter einbeziehen. Die Beteiligung der Gesellschafter spielt eine wichtige Rolle, wobei der WSF grundsätzlich einen Eigenkapitalbeitrag von 20 % anstrebt. Hierdurch soll sichergestellt werden, dass die Stabilisierung nicht einseitig durch den WSF erfolgt, sondern auch die Eigentümer einen wesentlichen Beitrag zur Stabilisierung leisten. Ein solcher Beitrag unterstreicht auch den Glauben der Gesellschafter an die Zukunft des Unternehmens. Die Ausgestaltung unterscheidet sich stark – die Beiträge reichen von Bareinlagen über Wandlungen von Fremd- in Eigenkapital bis hin zu Stundungen bestehender Ansprüche. In der Praxis waren die Gesellschafterbeiträge oft Gegenstand intensiver Verhandlungen zwischen WSF und Unternehmensseite bzw. Gesellschaftersphäre. Ein fehlender Gesellschafterbeitrag kann auch dazu führen, dass Stabilisierungsmaßnahmen nicht gewährt werden. Zusammengenommen ergibt die WSF-Zinslogik, dass die Zinsen z. B. bei niedriger Verschuldung, 20 %-Eigenanteil, krisenfestem Geschäftsmodell und sicherer Perspektive nur geringfügig oberhalb der EU-Mindestzinssätze liegen. Demgegenüber verlangt der WSF von hochverschuldeten Unternehmen mit krisenanfälligerem Geschäftsmodell höhere Zinssätze. Um einen Anreiz zu einer schnellen Rückzahlung der Beiträge zu setzen, ist der Zinskupon über die Zeit ansteigend ausgestaltet. Zudem wird bei vorzeitiger Rückzahlung keine Vorfälligkeitsentschädigung fällig.

Schließlich haben die unterstützten Unternehmen während der Laufzeit Berichtspflichten und diverse Auflagen einzuhalten, z. B. Verbote von Boni- oder Dividendenzahlungen, aber auch Maßnahmen gegen Wettbewerbsverzerrung. Um sicherzustellen, dass WSF-Mittel zielgerichtet eingesetzt werden, vereinbart der WSF mit den Unternehmen teilweise das „Ringfencing" – ein Konzept, das verhindert, dass WSF-Mittel verbundenen Gesellschaften in Drittländern zugutekommen. Durch diese Maßnahmen soll individuell sichergestellt werden, dass die WSF-Mittel ausschließlich der Unternehmensstabilisierung dient. Allerdings strebt der WSF keine Kontrolle des operativen Geschäfts an und übt keinen Einfluss auf die Entscheidungen der Geschäftsführung aus. Insgesamt setzt der WSF durch Auflagen und aufsteigende Zinskupons Anreize, die einen möglichst schnellen Ausstieg des Staates sicher-

7 Der WSF-Ausschuss ist ein interministerieller Ausschuss. Er ist besetzt mit je einem/einer Vertreter:in des Bundeskanzleramts, der Bundesministerien der Finanzen, für Wirtschaft und Klimaschutz, für Arbeit und Soziales, für Justiz und für Digitales und Verkehr. Dem WSF-Ausschuss gehören als beratende Mitglieder darüber hinaus je ein/e Vertreter:in der Kreditanstalt für Wiederaufbau (KfW) sowie der Bundesrepublik Deutschland Finanzagentur GmbH an.

8 Unternehmen, die sich bereits zum 31. Dezember 2019 in (wirtschaftlichen) Schwierigkeiten befunden haben (EU-Definition) und diesen Status danach nicht wieder überwunden haben.

stellen sollen. Rekapitalisierungen sollen grundsätzlich nach sechs Jahren, spätestens aber nach zehn Jahren beendet werden.

### Beteiligte Institutionen und operative Durchführung

Das Bundeswirtschaftsministerium trifft die Entscheidung über die Stabilisierungsmaßnahme im Einvernehmen mit dem Bundesfinanzministerium. Je nach Art und Umfang der Maßnahme werden auch der WSF-Ausschuss oder die Kreditanstalt für Wiederaufbau (KfW) eingebunden (§ 20 StFG und § 2 Wirtschaftsstabilisierungsfonds-Übertragungsverordnung). Die bewilligten Maßnahmen werden durch die Finanzagentur verwaltet (§ 18 Abs. 1 Satz 1 StFG), wobei die Verwaltung nicht nur die Erstellung sowie den Abschluss der Verträge, sondern auch die Koordination der Unternehmensberichte umfasst. Die Finanzagentur untersteht hinsichtlich der Wahrnehmung dieser Aufgaben der Rechts- und Fachaufsicht des Bundesfinanzministeriums, das diese im Einvernehmen mit dem Bundeswirtschaftsministerium ausübt (§ 18 Abs. 1 Satz 3 StFG).

Die EU-Kommission hat dem WSF eine Beihilfegenehmigung für Maßnahmen bis 250 Mio. Euro erteilt. Darüber hinausgehende Stabilisierungsmaßnahmen müssen separat bei der Kommission notifiziert werden. In der Regel müssen die beihilferechtlichen Vorgaben der WSF-Genehmigung bzw. des befristeten Rahmens für staatliche Beihilfen zur Stützung der Wirtschaft der KOM vom 19. März 2020 (Temporary Framework)[9] eingehalten und entsprechende Auflagen, beispielsweise hinsichtlich der Vergütung des Managements, in die Verträge aufgenommen werden. Die Überwachung der Auflagen liegt in erster Linie in der Verantwortung des Bundes. Bei besonders umfangreichen Maßnahmen setzt die Kommission jedoch zusätzlich einen Monitoring Trustee (Beobachtungstreuhänder) ein, der die Einhaltung der Auflagen überwacht.

## Übersicht Branchen/Volumen

Im Startjahr 2020 haben 110 Unternehmen Interesse am WSF bekundet. Allerdings stammten viele Interessenbekundungen aus der Anfangszeit des WSF, zu der Unternehmen beispielsweise vorsorglich bei mehreren vorhandenen bzw. sich im Aufbau befindenden Programmen Interesse bekundeten. Ein Großteil dieser Interessenbekundungen wurde nicht weiterverfolgt, oftmals weil andere Programme in Anspruch genommen werden konnten oder die Zugangsvoraussetzungen nicht erfüllt waren. Im zweiten Pandemiejahr 2021 sind 24 weitere Interessenbekundungen eingegangen, überwiegend in der ersten Jahreshälfte. 15 der 24 Interessenbekundungen mündeten in konkreten Anträgen. Sämtliche WSF-Interessenbekundungen der Jahre 2020 und 2021 mündeten in 25 bewilligten Anträgen von 21 Unternehmen. Eine Betrachtung nach Branchen zeigt, dass die bisher stabilisierten Unternehmen schwerpunktmäßig aus dem verarbeitenden Gewerbe, dem Handel und der Tourismus- bzw. Reisebranche stammen – also aus den Branchen, die entweder stark von Lockdown-Maßnahmen oder von coronainduzierten Lieferkettenproblemen betroffen waren.

Bis zum 31.12.2021 hat der WSF 21 Unternehmen mit zusammengenommen 8,8 Mrd. Euro stabilisiert (Finanzagentur, 2022). Hiervon haben die Unternehmen bislang 5,5 Mrd. Euro gezogen und bereits 2,4 Mrd. Euro zurückgezahlt. 2022 hat der WSF bislang zusätzliche Mittel in Höhe von 220 Mio. Euro an Galeria Karstadt Kaufhof zur Verfügung gestellt.[10] Knapp 90% des insgesamt bewilligten Volumens entfällt auf die drei Unternehmen Deutsche Lufthansa (5.847,1 Mio. Euro), TUI (1.241 Mio. Euro) und FTI Touristik (603 Mio. Euro). Darüber hinaus haben 15 mittelständische Unternehmen Unterstützungsmaßnahmen des WSF erhalten. Die kleinste Einzelmaßnahme betrug 3,8 Mio. Euro. Insgesamt wurden vier Unternehmen mehrere Unterstützungsmaßnahmen des WSF gewährt. Bei allen bewilligten Anträgen des WSF handelt es sich um Rekapitalisierungen (keine Garantien). Der Großteil dieser Rekapitalisierungen sind hybride Finanzinstrumente wie Nachrangdarlehen oder Stille Beteiligungen. Der WSF ist bislang nur eine offene Beteiligung eingegangen.

## Zwischenbilanz und Ausblick

Der WSF ist Teil des staatlichen Rettungspakets der Coronakrise und richtet sich an Unternehmen mit besonderer volkswirtschaftlicher Bedeutung. Die Entfaltung der Förderlandschaft bzw. das Entstehen neuer Instrumente ist eine Folge des dynamischen pandemischen, wirtschaftlichen und politischen Prozesses. Da das Instrumentarium zur Bekämpfung der wirtschaftlichen Auswirkungen der Coronakrise erst sukzessive auf- und ausgebaut wurde, haben zunächst viele Unternehmen Interessenbekundungen an den WSF gerichtet. Diese wurden oft nicht weiterverfolgt, weil entweder mit der KfW günstigere Fremdkapitalangebote zur Verfügung standen oder zwischenzeitlich nicht rückzahlbare Zuschussprogramme (Überbrückungshilfen sowie November-/Dezemberhilfen) Gestalt annahmen. In Einzelfällen wurde der WSF als Brückenfinanzierung herangezogen. Daraus

9 Mitteilung C(2020) 1863 der Kommission vom 19. März 2020 „Befristeter Rahmen für staatliche Beihilfen zur Stützung der Wirtschaft angesichts des derzeitigen Ausbruchs von COVID-19" (ABl. C 91 I vom 20.3.2020, 1): ABl. 2020 C 91, I/1, geändert durch ABl. 2020 C 112, I/1, ABl. 2020 C 164, 3, ABl. 2020 C 218, 3 und ABl. 2020 C 340 I/1, ABl. 2021 C 034, 6 und ABl. 2021 C 473, 1.

10 Zudem werden 30 Mio. Euro des im Februar 2021 gewährten Nachrangdarlehens für Galeria Karstadt Kaufhof in die Stille Einlage überführt, wodurch sich ein Gesamtvolumen von 250 Mio. Euro ergibt.

ergibt sich ein erheblicher Aufwand für die Unternehmen, aber auch für die Stellen der öffentlichen Hand, die über die Unterstützungsmaßnahmen zu entscheiden haben. Einigen Unternehmen war bei Antragsstellung unklar, dass der WSF eine risiko- bzw. marktgerechte Verzinsung verlangt, um die reguläre Finanzierung über den Kapitalmarkt nicht zu verdrängen. Schließlich haben die Beihilfeauflagen eine für Unternehmen teils abschreckende Wirkung entfaltet.

Trotz der nach absoluten Fallzahlen kleinen Zahl stabilisierter Unternehmen ist der WSF ein erfolgreicher und wichtiger Baustein der Krisenarchitektur. Denn Unternehmen wie die Deutsche Lufthansa hätten anderweitig nicht gerettet werden können. Zudem handelt es sich stets um Unternehmen mit besonderer Bedeutung für die deutsche Wirtschaft mit einer großen Zahl an Arbeitsplätzen, einer regionalpolitischen oder technologischen Bedeutung. Ihre Stützung hat damit Zweitrundeneffekte für die gesamte Wertschöpfungskette von Zulieferern, Kundschaft und Unternehmensbeteiligungen. So hat der WSF nicht nur einen großen Teil des Flug- und Reisemarkts in Deutschland gestützt, sondern auch Arbeitsplätze in strukturschwachen Regionen langfristig erhalten. Außerdem verdeutlicht der WSF die Wirksamkeit einer fondsgetriebenen Maßnahme. Die Instrumente des WSF wurden zwar bis jetzt selten in der deutschen Förderlandschaft eingesetzt, haben sich jedoch bewährt. Die flexiblen eigenkapitalähnlichen Hybridinstrumente des WSF können gezielt auf spezifische Bedarfe zugeschnitten werden.

Allerdings ist ein Instrument wie der WSF aufgrund der aufwändigen Einzelfallprüfung (Risikobewertung, Strukturierung, Verwaltung/Monitoring) nicht beliebig einsetz- oder skalierbar, da hier ein großer Zeit- und Kostenaufwand auf Unternehmens- und Vergabeseite entsteht. Aufwand und Ertrag sollten in einem für beide Seiten sinnvollen Verhältnis stehen: Standardisierte Hilfen oder Zuschüsse können bei kleineren Vorhaben schneller und günstiger geprüft werden. Fondslösungen wie der WSF empfehlen sich daher insbesondere für hohe Bedarfe oder Spezialfälle, die individuell zugeschnitten werden müssen. Die Geltungsdauer des WSF ist begrenzt: WSF-Stabilisierungsmaßnahmen, die stets der Bewältigung der Pandemiefolgen dienen, können auf der Basis der geltenden gesetzlichen Grundlage und des beihilferechtlichen Rahmens noch bis zum 30. Juni 2022 gewährt und bis zum 30. April 2022 beantragt werden.

Aber die Erfahrungen mit dem WSF könnten auch bei der Bewältigung wirtschaftlicher Transformationsprozesse zur Erreichung der Klimaneutralität hilfreich sein. So könnte ein staatlicher Fonds, der Unternehmen Nachrangkapital mit klarer Zweckbindung an Klimaschutz- und Transformationsvorhaben gewährt, ein Baustein bei der Transformation der Wirtschaft sein. Denn die Stärkung der Eigenkapitalbasis ermöglicht auch eine bessere Finanzierung am Fremdkapitalmarkt. Erfolgreiche Kapitalerhöhungen, wie sie z. B. von Lufthansa oder TUI während der WSF-Stabilisierungen durchgeführt wurden, verdeutlichen diesen Effekt. Möglichkeiten zur Umsetzung einer solchen Fondslösung werden derzeit intensiv im Wirtschaftsministerium diskutiert. Primäre Zielgruppe wären Unternehmen, die Transformationsvorhaben in Eigenregie nicht oder nur in geringem Maße umsetzen können – z.B. die sehr teure Umstellung auf klimaneutrale Produktion. Außerdem wird zurzeit geprüft, ob durch die Verstetigung des WSF ein permanent passiv bestehendes und kurzfristig aktivierbares Kriseninstrument zur Unternehmensstabilisierung geschaffen werden könnte. Beihilferechtliche und fiskalpolitische Erwägungen müssten dafür weiterhin im Detail geprüft werden. Anders als zu Beginn der Coronakrise liegt mit dem WSF mittlerweile allerdings eine Blaupause für künftige Modelle zur Stärkung der Eigenkapitalbasis vor.

**Title:** ***The Economic Stabilisation Fund – An (Interim) Assessment***
